# Quantifying the Protection of Activating and Inhibiting NK Cell Receptors during Infection with a CMV-Like Virus

**DOI:** 10.3389/fimmu.2014.00020

**Published:** 2014-01-30

**Authors:** Paola Carrillo-Bustamante, Can Keşmir, Rob J. de Boer

**Affiliations:** ^1^Theoretical Biology and Bioinformatics, Department of Biology, Utrecht University, Utrecht, Netherlands

**Keywords:** NK cell receptors, evolution, CMV infection, models, theoretical, agent-based modeling

## Abstract

The responsiveness of natural killer (NK) cells is controlled by balancing signals from activating and inhibitory receptors. The most important ligands of inhibitory NK cell receptors are the highly polymorphic major histocompatibility complex (MHC) class I molecules, which allow NK cells to screen the cellular health of target cells. Although these inhibitory receptor–ligand interactions have been well characterized, the ligands for most activating receptors are still unknown. The mouse cytomegalovirus (MCMV) represents a helpful model to study NK cell-driven immune responses. Many studies have demonstrated that CMV infection can be controlled by NK cells via their activating receptors, but the exact contribution of the different signaling potential (i.e., activating vs. inhibiting) remains puzzling. In this study, we have developed a probabilistic model, which predicts the optimal specificity of inhibitory and activating NK cell receptors needed to offer the best protection against a CMV-like virus. We confirm our analytical predictions with an agent-based model of an evolving host population. Our analysis quantifies the degree of protection of each receptor type, revealing that mixed haplotypes (i.e., haplotypes composed of activating and inhibiting receptors) are most protective against CMV-like viruses, and that the protective effect depends on the number of MHC loci per individual.

## Introduction

Natural killer (NK) cells contribute to the host immune response by recognizing and killing viral-infected and tumor cells (1). Their activity is controlled by balancing signals from a vast repertoire of activating and inhibiting receptors enabling them to distinguish healthy from unhealthy cells (2). The most important ligands for inhibitory NK cell receptors (iNKR) are MHC class I molecules on other cells. An infected cell may have lower MHC expression, altering the binding with inhibitory receptors, disrupting the balance of signals, and allowing for NK cell activation. The mechanism by which NK cells attack MHC class I deficient cells was coined by Kärre et al. as “missing-self” detection (3).

There are several NKRs that contribute to missing-self detection. In humans, for example, the inhibitory receptor CD96/NKG2A binds to complexes of the human leukocyte antigen-E (HLA-E), which presents peptides derived from the leader sequences of HLA-A, -B, and -C molecules (4, 5). Both the receptor and the ligand are highly conserved in these inhibitory interactions, and the down-stream effects are remarkably similar across individuals (6). The killer immunoglobulin-like receptors (KIRs) also contribute to monitor abnormalities in MHC class I expression on cell surfaces. In contrast to the CD96/NKG2 superfamily, KIRs are highly polygenic and polymorphic, exhibit both inhibitory and activating potential, and bind to the highly polymorphic HLA-A, -B, and -C molecules (7–9). Consequently, the interactions between KIRs and classical HLA-class I molecules are very diverse (10). Thus, humans have two types of NKRs, one conserved and one highly diverse, performing seemingly the same function.

Humans are not the only species that have an expanded and polymorphic NKR gene complex. During mammalian radiation, many different species have diversified alternative NKR gene families recognizing MHC class I. This example of convergent evolution includes three gene families from two structurally unrelated superfamilies: KIRs, the CD94/NKG2, and the Ly49 (11). Higher primates have expanded their KIR genes (12); a group of lower primates have expanded NKG2 (13), whereas rodents and equids have expanded Ly49 (14, 15). These alternative genetic strategies illustrate the evolutionary complexity of these systems, and suggest that an expanded NKR gene complex is beneficial for survival. But, if conserved inhibitory receptor–ligand interactions (such as NKG2A–HLA-E in humans) are capable to successfully detect missing-self, why have several NKR families evolved to become polygenic and polymorphic? Even more intriguing, why have they evolved receptors with activating potential?

In humans, some activating NKRs (aNKRs) are associated with the disease outcome of viral infections and malignancies (16). For example, in combination with HLA–Bw4, the activating KIR3DS1 has been associated with a delayed progression to AIDS in HIV-1 infected individuals (17, 18). KIR3DS1 has also been linked to an increased rate of spontaneous recovery after hepatitis B infections (19), a reduced risk of developing hepatocellular carcinoma in patients infected with HCV (20), and a reduced risk of Hodgkin’s lymphoma (21). Moreover, maternal activating KIRs are related to protection against several pregnancy disorders (22). But because only a few ligands for activating KIRs have been identified so far, the exact mechanisms underlying the provided protection in humans remain puzzling.

Studies in mice have revealed important insights into the role of aNKR during viral infections (23, 24). Viruses like the mouse cytomegalovirus (MCMV) down-regulate the expression of MHC class I molecules from the cell surface to escape T cell response, and may additionally code decoy MHC molecules (m157) that can inhibit NK cell activation (23). Mouse strains that are resistant to MCMV carry the activating Ly49H gene, which binds with high affinity to the MHC-like viral protein m157. In contrast, mice susceptible to MCMV lack the activating gene but carry the inhibiting receptor Ly49I, which also binds strongly to the m157 protein. The activating Ly49H emerged from an inhibitory counterpart (25), suggesting that the evolution of an aNKR was due to the immune pressure induced by the “MHC decoy” m157 during CMV infection (26, 27).

Although these studies shed light into the importance of NKR in general, the specific contribution of activating and inhibitory receptors to the NK cell response is still unknown. We previously studied the evolution of KIR diversity in a human population infected with CMV-like viruses by using a computational agent-based model (28). We showed that iNKRs require sufficient specificity to protect populations against viruses evolving MHC-like molecules, and that diversity in the NK cell genetic complex evolves as a result of the required discrimination between self-MHC molecules and viral decoy molecules. Here, we also consider aNKRs, and develop a probabilistic model to quantify the optimal specificity of inhibitory and activating NKRs needed to render maximal protection against CMV-like viruses. We also analyze the effect of mixed haplotypes (i.e., composed of aNKR and iNKR) on protection, and confirm the expectations of the probabilistic model with an agent-based computational model. Our studies reveal that mixed haplotypes composed of specific activating and inhibitory NKRs render high protection against CMV-like viruses encoding for decoy molecules, and that the protective effect depends on the number of MHC loci per individual.

## Results

We analyze the effect of the specificity of activating and inhibitory NKRs on the detection of a virus presenting MHC-like molecules with a simple probabilistic model. Our model estimates the chance of protection *P*, i.e., the probability of a host detecting an infection by NK cells, as a function of the haplotype size, specificity (i.e., the probability *p* of recognizing any random MHC molecule), and number of MHC loci.

The responsiveness of NK cells (i.e., their ability to discriminate cells with normal MHC expression from those lacking MHC) is regulated by a process called “education” or “licensing” taking place during NK cell development (29). During this process, the interactions of iNKRs with their MHC ligands render the NK cells with functional competence (13, 29, 30). To prevent NK cell-related autoimmunity, activating receptors also participate in the education process, where the chronic exposure of aNKR ligands during development results in hyporesponsive NK cells (31, 32).

**Figure 1:**
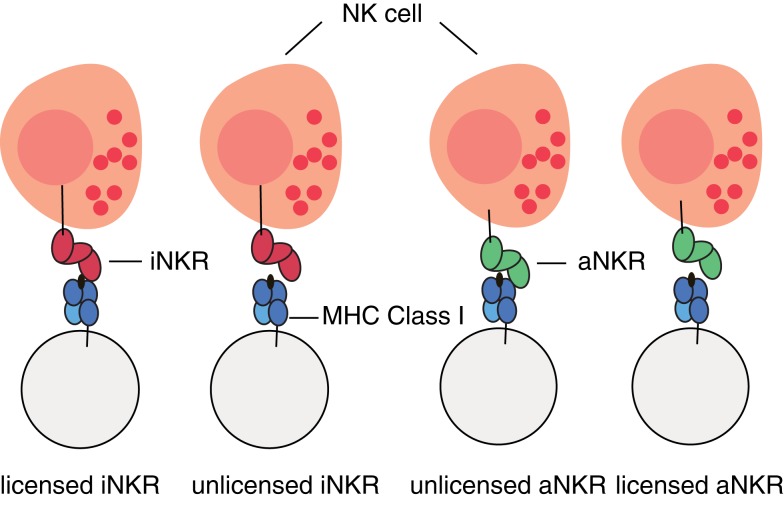
**Cartoon of NK cell education**. iNKRs (represented in red) recognizing at least one of the MHC molecules per individual will become licensed. In contrast, aNKRs (depicted in green), which do not recognize *any* of the host’s MHC molecules will become licensed.

For simplicity, we do not model individual NK cells, each expressing a random set of tuned receptors. We rather consider for each individual a global repertoire of receptors, which have the potential to license NK cells. Henceforth, we will refer to these receptors as “licensed” receptors. We mimic the MHC-dependent education process during NK cell development by creating a repertoire of NKRs composed of iNKRs that recognize at least one of the MHC molecules of the host, and of aNKRs that recognize none of the MHC molecules of the host (Figure 1). By considering a global repertoire, we assume that there will be at least one subset of NK cells expressing at least one of the “licensed” NKRs. Upon infection, we consider only those NK cell subsets having licensed receptors. If these can successfully detect the virus, they will become activated, expand, and protect (see Appendix for a full discussion). Therefore, only the licensed repertoire of NKRs is allowed to participate in the immune response.

Whether a decoy protein allows a virus to successfully escape the NK response, i.e., whether that individual is protected against the infection, depends on the receptor type and on the receptor specificity. iNKRs that bind the decoy molecule cannot detect missing-self and are “fooled” by the decoy. Conversely, aNKRs binding the foreign decoy protein can specifically recognize the infection and therefore protect the host. With this model, we quantify the contribution of each receptor type and its specificity to the detection of CMV-like viruses.

### Inhibitory and activating NK cell receptors differ in the protection level they provide

There are two crucial processes for a single iNKR to detect virus evolving MHC-like molecules. First, iNKRs have to be licensed during the NK cell education to become fully functional during an immune response. Second, iNKRs should not bind decoy molecules upon infection. Because, in our model, iNKRs are only licensed if they recognize at least one of the MHC molecules in their host, and decoy molecules are similar to self-MHC molecules, iNKRs face the challenge of distinguishing self-MHC molecules from foreign decoy molecules. We previously demonstrated that this challenge can be solved by evolving sufficiently specific iNKRs (28). In that study, we defined specificity as the probability (*p*) of any NKR to recognize a random MHC molecule in the population. Herewith, degenerate receptors (i.e., with *p* = 1) are able to recognize all MHC molecules in the population, whereas specific receptors (i.e., with *p* ∼ 0) recognize only a small fraction of them. Since the exact relation between ligand–receptor binding affinity and signaling potential remains unknown, we do not consider different binding affinities here, and we model discrete MHC–NKR interactions.

To study whether there is an optimal specificity for which iNKRs are not inhibited by such “decoy viruses,” we calculated the probability of licensed iNKRs detecting the infection. A single iNKR becomes licensed with a probability $q_{I}=1-{\left(1-p_{I}\right)}^{2N_{\mathrm{MHC}}},$ where *p*_I_ describes the specificity (i.e., the probability of any iNKR to recognize any MHC in the population), and *N*_MHC_ the number of MHC loci per individual. The probability of a haplotype composed of *N*_iNKR_ to have exactly licensed iNKRs is given by the binomial distribution as follows:

(1) $$P\left(\mathrm{iNK}R_{\mathrm{licensed}}=\ell \right)=\left(\begin{array}{c} N_{\mathrm{iNKR}}\\ \ell \end{array}\right){\left(1-q_{I}\right)}^{N_{\mathrm{iNKR}}-\ell }q_{I}^{\ell }.$$

To successfully detect a decoy virus, none of the licensed iNKRs should bind the decoy molecule. Thus, the overall probability of detecting the infection is determined by the chance that none of the licensed iNKRs recognizes a decoy molecule, and can be calculated by:

(2) $$P_{I}\left(\mathrm{detection}\right)=\sum_{\ell =1}^{N_{\text{iNKR}}}\;\left(\begin{array}{c} N_{\mathrm{iNKR}}\\ \ell \end{array}\right){\left(1-p_{I}\right)}^{\ell }{\left(1-q_{I}\right)}^{N_{\mathrm{iNKR}}-\ell }q_{I}^{\ell }.$$

**Figure 2:**
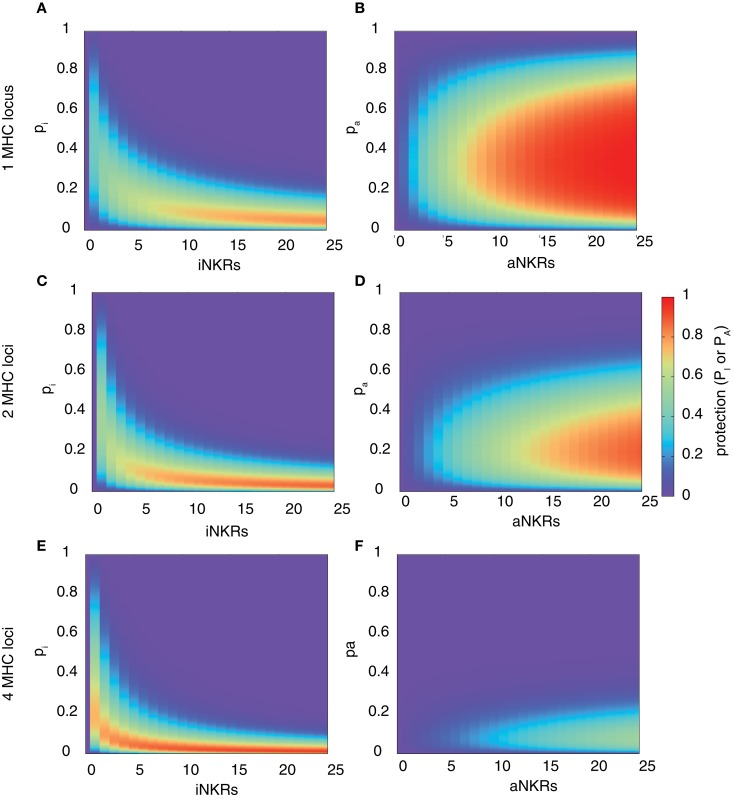
**Range of protection differs between iNKRs and aNKRs**. The heatmaps show the protection level as the probability of detecting the infection with a virus expressing a decoy molecule to mask MHC down-regulation. In the left column, the protection for individuals carrying only iNKRs [calculated by equation (2)] is shown, whereas in the right column, the protection for individuals carrying only aNKRs [calculated by equation (3)] is depicted. The protection level is shown in the color bar from highest (red) to lowest (blue). *p_i_* and *p_a_* correspond to the specificity of iNKR and aNKR, respectively. **(A,B)** The protective range for iNKRs is small and skewed toward a large haplotype size and high specific values. In contrast, aNKRs offer a broad range of protection for intermediate specificity values and a smaller haplotype size. Calculations were done with 1 MHC locus **(A,B)**, 2 MHC loci **(C,D)**, and 4 MHC loci **(E,F)**.

Our analysis confirms that for any haplotype size, there is an optimal specificity. For *N*_iNKR_ ≤ 25, our model predicts a maximal level of protection (i.e., *P*_I_ = 0.85), which can only be obtained with high specificity values (*p*_I_ ≤ 0.2) and a large number of genes per haplotype (*N*_iNKR_ ≥ 20) (Figure 2A).

A host with degenerate iNKRs (e.g., *p*_I_ ≥ 0.8) has a large repertoire of licensed iNKRs. But because of the low specificity, the iNKRs within that individual are expected to also recognize any foreign decoy molecule as self, offering no protection. In contrast, when iNKRs are specific (e.g., *p*_I_ ≤ 0.2) the repertoire of licensed iNKRs per individual is lower, but if there are several genes per haplotype, the chance of having at least one licensed specific iNKR increases. Due to their high specificity, it is unlikely for a licensed iNKR to also recognize a foreign decoy molecule, impeding the virus to escape the NK immune response. Therefore, an infection with a decoy virus can be controlled with a probability of at least 70% in a haplotype composed of more than 10 iNKRs when *p*_I_ ≤ 0.25. Thus, the probability of detecting the virus increases with both a higher specificity and a larger number of genes per haplotype (i.e., *N*_iNKR_). This confirms our previous results, suggesting that large haplotypes composed of non-overlapping specific iNKRs are most protective (28).

We next developed a model considering only aNKRs. Similar to the iNKRs, the two crucial processes for an aNKR to detect the virus depends on the probability of becoming licensed and recognizing the decoy molecule as a foreign antigen. However, the licensing process is almost opposite between aNKRs and iNKRs. An aNKR becomes licensed if it does not recognize any MHC molecule within an individual. The probability of a single aNKR to become licensed is therefore described by $q_{A}={\left(1-p_{A}\right)}^{2N_{\mathrm{MHC}}},$ where *p*_A_ is the specificity of an aNKR. Opposite to an iNKR, an aNKR detects a “decoy virus” if it binds the MHC decoy. Thus, the overall probability of protection in this case is determined by the chance of at least one licensed aNKR binding the decoy molecule, and is given by:

(3) $$\begin{array}{llll} P_{A}\left(\mathrm{detection}\right) & =\sum_{\ell =1}^{N_{\text{aNKR}}}\;\left(\begin{array}{c} N_{\mathrm{aNKR}}\\ \ell \end{array}\right)\left(1-{\left(1-p_{A}\right)}^{\ell }\right)\; & & \;\\ & \;\times {\left(1-q_{A}\right)}^{N_{\mathrm{aNKR}}-\ell }q_{A}^{\ell },\; \end{array}$$

where *N*_aNKR_ is the number of aNKRs per haplotype.

This model reveals that there is again an optimal specificity, and the protection range for aNKR is much broader than that for iNKR, covering also less specific receptors (i.e., 0.1 ≤ *p*_A_ ≤ 0.7) (Figure 2B). In these cases, the optimal protection (i.e., *P*_A_ = 1) is obtained with haplotypes composed of 12 genes, having intermediate specificity values (0.2 ≤ *p*_A_ ≤ 0.65). To avoid self-reactivity, aNKRs become licensed only if they fail to recognize all self-MHC molecules. Additionally, an aNKR must recognize foreign MHC-like molecules to detect the infection. Therefore, the challenge for an aNKR is opposite to that of an iNKR, since it must recognize foreign antigens but not self-MHC molecules. A degenerate aNKR will recognize every decoy in the population but it will never become licensed. As a result, the optimal protection is reached in large haplotypes composed of aNKRs with intermediate specificity.

Note that we consider individuals to be heterozygous for all MHC loci. Allowing individuals to be homozygous in some MHC loci does not qualitatively change our results on specificity and protection, since MHC homozygosity has only a mild effect on the number of licensed receptors, ℓ (results not shown).

### The protection level depends on the number of MHC loci

Above, we considered only one MHC locus per individual as a representation of HLA-C as the main identified ligand for inhibitory KIRs. However, HLA-A and -B molecules have also been identified as KIR ligands, and HLA-E is the ligand for CD94/NKG2A. Therefore, we expanded our model to consider two MHC loci per individual. The distribution of protection levels is similar to the model with one MHC locus, showing a small protective area for individuals carrying only iNKRs (Figure 2C), whereas individuals carrying aNKRs have a broader protective range (Figure 2D). However, the area of maximal protection is skewed in both cases. Because iNKRs have to recognize at least one self-MHC molecule to become licensed, the chance of having several licensed NKRs per haplotype increases by having 2 MHC loci (and thus 4 MHC molecules per heterozygous individual). Therefore, a high protection (e.g., *P*_I_ ≥ 0.85) can be reached already with a smaller haplotype, e.g., one composed of at least 11 iNKRs.

In contrast, the probability of an aNKR to become licensed decreases with 2 MHC loci because aNKRs should not recognize *any* of the MHC molecules within an individual. Consequently, the protection with aNKRs reaches high values (i.e., *P*_A_ ≥ 0.85) only with large haplotypes composed of at least 20 genes and the optimal protection level (*P*_A_ = 1) is never obtained. Thus, the protection of aNKRs is highly dependent on the number of MHC molecules per individual.

With even higher MHC complexity, i.e., by increasing the number of MHC loci per individual to 4, fewer iNKRs are sufficient to successfully clear the infection (Figures 2E,F). Because of the education process in our model, hosts with 4 MHC loci have a much larger licensed iNKR repertoire compared to individuals having 1 MHC locus. These hosts reach the maximal protection already with a haplotype size of 4 receptors. Even for lower haplotype sizes, a good protection level (i.e., 0.3 ≤ *P*_I_ ≤ 0.7) can be reached at lower specificity values (*p*_I_ ≤ 0.35) (Figure 2E). This effect was further increased when considering 8 MHC loci per individual, where the maximal protection was reached with only one specific iKIR (results not shown).

However, an expanded MHC haplotype is disadvantageous for individuals having only aNKRs. Because in our model the licensing process is more difficult with a higher number of MHC molecules, little protection can be provided. The infection can be controlled with a maximal probability of 50 and 35% in individuals with 4 (Figure 2F) and 8 MHC loci (results not shown), respectively.

Taken together, these results show that aNKRs provide little protection against a virus evolving MHC decoy proteins in individuals having several MHC loci, and that a contracted haplotype of iNKRs is already protective when the MHC complexity increases.

### Viral detection is maximal in mixed haplotypes

To predict the combined protection of activating and inhibitory NKRs, we expanded our model and considered mixed haplotypes, i.e., haplotypes composed of both iNKRs and aNKRs. We predict the combined probability of detecting the virus as follows:

(4) $$P=1-\left(1-P_{I}\right)\left(1-P_{A}\right).$$

**Figure 3:**
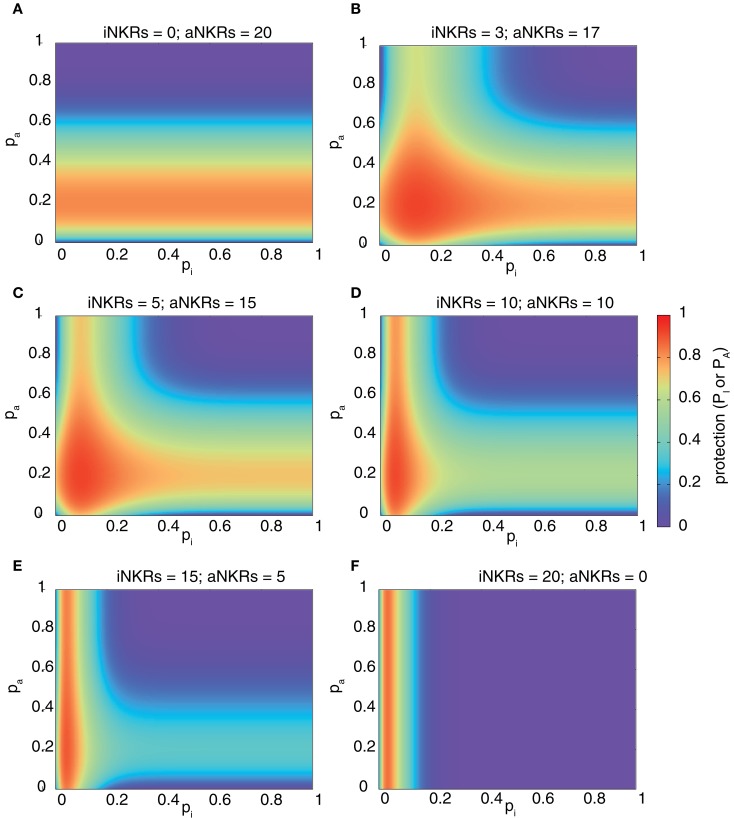
**Mixed haplotypes render highest protection**. The heatmaps show the protection level as the probability of detecting the infection for haplotypes composed of iNKRs and aNKRs [as calculated by equation (4)]. The protection level is shown in the color bar from highest (red) to lowest (blue). **(A–F)** Show the NKR haplotype composition for different fractions of iNKR and aNKR. Considering a haplotype size of *N*_NKR_ = 20, we first modeled haplotypes composed of aNKRs only **(A)**, and reduced their number, while increasing the number of iNKRs **(B–E)**, until we obtained a haplotype composed only of iNKRs **(F)**. We here consider 2 MHC loci (i.e., *N*_MHC_ = 2).

We computed the protection in hosts carrying two MHC and 20 NKR loci, and varied the fraction of aNKRs in the NKR haplotype, while keeping the total number of loci constant. The best protection is reached in mixed haplotypes (Figure 3). As seen above, haplotypes with aNKRs only provide protection (i.e., 0.5 ≤ *P* ≤ 0.8) for intermediate specificity values 0.15 ≤ *p*_A_ ≤ 0.4 (Figure 3A). With increasing number of iNKRs per haplotype, the protection reaches higher values (approaching *P* = 1) (Figures 3B,C), covering a larger range of specificity values and having a skewed distribution toward more specific inhibitory and activating receptors. A large number of iNKRs per haplotype reduces the contribution of aNKRs, and therefore the latter can have low specificity values without affecting the protection level (Figures 3D,E). Note that the area of high protection shrinks when the fraction of aNKRs is decreased, where maximal protection can only be achieved for extremely high specificities (i.e., *p*_I_ ≤ 0.1) (Figure 3F).

These results depend on a similar manner on the number of MHC loci per individual as those shown in Figure 2, with aNKRs having a lower protective effect with increasing MHC loci number (results not shown). Therefore, we conclude that the maximal protection against CMV-like viruses is easier to achieve in mixed haplotypes.

### Agent-based model of activating and inhibitory NKRs

Our probabilistic model allows us to quantify the expected protection, given a certain number of aNKRs and iNKRs. However, it is not clear whether a population with evolving NKRs would find the same basin of attraction for the specificity (i.e., *p*) when infected with a CMV-like virus.

To study the evolution of NKR specificity in populations infected with a CMV-like virus, we developed an agent-based model similar to the one published in Ref. (28) (for a detailed description of the model, see Materials and Methods). Briefly, our model considers a human population infected with a non-lethal herpes-like virus causing chronic infections. The hosts carry a diploid genome with one or two MHC loci and ten NKR loci. We consider 15 MHC alleles per locus (mimicking the common HLA-B and -C alleles in the European populations) and this polymorphism is kept constant throughout the entire simulation (i.e., we do not allow for mutation of the MHC genes). The initial NKR haplotype consists of ten different genes, and all individuals are homozygous for the same NKR haplotype.

Upon birth, novel receptors can be created, allowing for evolution within the NKR gene complex. Each new receptor comes with a randomly chosen receptor type (i.e., either inhibitory or activating) and a randomly chosen specificity value (corresponding to 0 ≤ *p* ≤ 1, see Materials and Methods). Receptors are so specific that they are unable to recognize any MHC in the population will never be functional, and are considered to be pseudogenes. Thus, haplotypes expand by acquiring receptors with novel *p* values and signaling potential, but can also contract due to the accumulation of pseudogenes.

In this agent-based model, we also mimic the MHC-dependent NK cell education process (Figure 1). We remove iNKRs which fail to recognize any MHC molecule within an individual from the licensed repertoire. Similarly, those aNKRs capable of recognizing self-MHC molecules are deleted from the licensed repertoire. Only the licensed NKRs are able to participate during the immune response.

Infection of a host starts with a short acute phase, after which the individual either recovers or becomes chronically infected. We consider one wild-type virus and several decoy viruses (1 decoy per MHC molecule in the population). We do not allow for superinfection nor co-infection, thus hosts can be infected with only one of the viruses. A decoy virus down-regulates the expression of all MHC molecules within an individual, and expresses an MHC-like molecule. Thus, every virus expressing a decoy molecule has the potential to escape the immune response of both T and NK cells. The evolution of decoy proteins is modeled by allowing the virus to adopt a randomly selected MHC molecule from its host. Therefore, each decoy protein is actually an MHC molecule.

**Figure 4:**
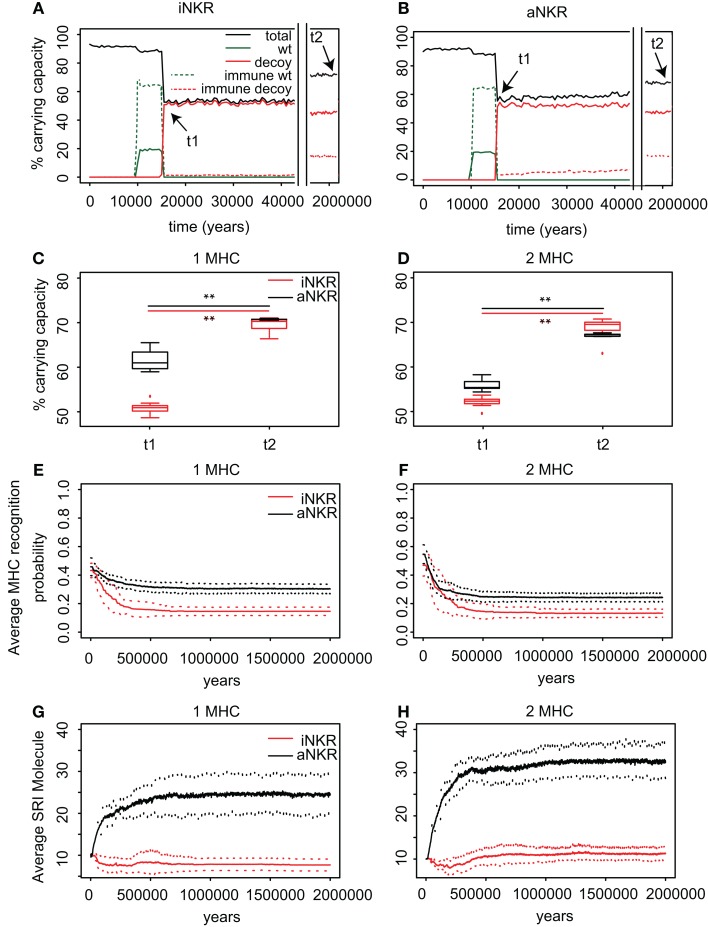
**Agent-based model confirms probabilistic model**. **(A)** A host population having only iNKRs was inoculated with a wild-type virus (green lines) after a period of *t* = 5000 years (green solid lines show the chronically infected individuals and the dashed lines the immune individuals). Ten thousand years after the initial epidemic (i.e., *t*_1_), we allowed for the evolution of decoy viruses (red lines). During the wild-type infection, most individuals recover (dashed green line). In contrast, almost none of the individuals are initially capable of clearing a CMV-like infection (red dashed line), resulting in a large decrease of the total population size (black line). **(B)** A host population having only aNKRs is initially better protected against decoy viruses, resulting in a higher fraction of the population clearing the infection, and a lower decrease of the total population size. **(A,B)** Show single representative simulations. **(C)** The average population size during the initial spread of decoy viruses (*t*_1_) is lower than that at the end of the simulations (i.e., *t*_2_ = 3 million years), indicating that over time, the populations learn to cope with the viral infection. Individuals in simulations considering only aNKRs (black) are initially better protected than those in simulations considering only iNKRs (red). In these simulations, all hosts carry only one MHC locus. **(D)** The initial advantage that aNKRs have over iNKRs receptors decreases in simulations considering two MHC loci per individual. **(E)** The probability of iNKRs recognizing any random MHC molecule in the population decreases over time (red line), indicating that more specific receptors are being selected for. In contrast, aNKRs (black line) do not evolve such high degree of specificity. **(F)** aNKRs evolve to become more specific in simulations where individuals have two MHC loci. **(G)** The degree of NKR polymorphism (expressed as the SRI score) increases in time, as a result of the evolved higher specificity. **(H)** SRI score in simulations considering two MHC loci. In **(C,D)**, the boxes represent the interquartile range, and the thick horizontal lines the median out of ten simulations (**represent *p* values < 0.005 and were calculated using the Mann–Whitney *U* test). In **(E–H)**, the solid lines represent the average out of ten simulations, and the dashed lines are the standard deviation.

The population is first inoculated with the wild-type virus, which can be typically cleared after the acute phase because of the implicit response of both T and NK cells. We model the immune response with one parameter describing the probability of clearing the infection. For the wild-type virus, this is set to *p_wt_* = 0.85 (Table 1), resulting in approximately 85% of the wild-type infections being cleared. Individuals clearing the infection become immune for a period *t*_i_ of 10 years. At steady state, approximately 20% of the population becomes chronically infected (Figures 4A,B; green solid lines), 65% become immune (Figures 4A,B; green dashed lines), and 5% are susceptible for infection. The immune escape of the decoy viruses is modeled by decreasing the clearance probability to zero (*p*_dec,1_ = 0, Table 1), which occurs if at least one of the licensed iNKRs or none of the aNKRs binds to the decoy molecule (Table 2). With this agent-based model, we can study the evolution of NKR specificity, and quantify the protection provided by activating and inhibitory receptors.

**Table 1:** **Parameters of the agent-based model**.

Parameter	Value
Time step	1 week
Simulation time	2 Million years
**HOST PARAMETERS^a^**	
Maximal population size, *N*_max_	5000 Individuals
MHC diversity	1–2 Loci, each with 15 alleles
Number of NKR loci	5–10
Bit string length	16 Bits^b^
Host mutation rate, μ	0.00005 Per gene per birth event
**INFECTION^c^**	
Infection state, *i*	1 (Acute), 2 (chronic)
Effect of viral load on the death rate, *VL_i_*	0.1 (For *i* = 1), 0.06 (for *i* = 2) per year
Probability of viral transmission during acute phase, *p_ac_*	0.85 Per contact
Probability of viral transmission during chronic phase, *p_ch_*	0.15 Per contact
Probability of clearing the wild-type virus, *p_wt_*	0.85
Success state of the decoy virus, *s*	0 (Successful), 1 (unsuccessful)
Probability of clearing the virus evolving decoy molecules, *p_dec_*_,_*_s_*	0 (For *s* = 0), 0.5 (for *s* = 1)
Immunity time, *t*_i_	10 years
Acute infection time, *t*_inf_	4 weeks
**VIRUS PARAMETERS**	
Virus mutation rate, $\mu_{v}$^d^	0.0001 Per week
**INITIAL CONDITIONS**	
Initial population size, *N*_init_	4500 Individuals
KIR initial diversity (SRI)	5–10 (1 Allele per locus)

*^a^The death and birth rate parameters are age-dependent and have been chosen according to a human population (33). For a full description of the age-dependency of birth and death rate, see Ref. (28)*.

*^b^The choice to use 16-bit strings represents a large enough theoretical repertoire of 65,536 sequences*.

*^c^The parameters used for the infection are chosen to maintain the epidemic. Changing the length of the acute phase or the probabilities of clearance do not affect our results on the evolution of the NKRs qualitatively (results not shown)*.

*^d^We manually switch on the mutation of the viruses at specific points in time, and after that the mutation rate determines the waiting time for the mutant to arrive. The mutant viruses appear in a short time scale and once the virus has spread in the population, mutation does not occur anymore. Since we analyze the genetic diversity long after the arrival of the virus, changes in mutation rate should not affect the outcome*.

**Table 2:** **Levels of protection against a decoy virus in the agent-based model**.

	No. of aNKR_binding_ = 0	No. of aNKR_binding_ > 0
No. of iNKR_binding_ = 0	*p_dec_*_,1_	*p_dec_*_,1_
No. of iNKR_binding_ > 0	*p_dec_*_,0_	*p_dec_*_,1_

*aNKR_binding_ and iNKR_binding_ refer to the number of iNKRs and aNKRs binding the decoy molecule, respectively. The receptors here are considered to be licensed*.

### Inhibitory receptors evolve higher specificity than activating receptors after a CMV-like infection

We first study the protection provided by iNKRs against a CMV-like virus. After 5000 years of infection with the wild-type virus, we allow for the emergence of decoy viruses. The initial specificity of the iNKRs is set to *p* ≈ 0.4 (see Materials and Methods). The decoy viruses spread easily among individuals carrying only iNKRs, resulting in a high fraction of chronically infected individuals (Figure 4A; red solid lines). Moreover, almost none of the hosts is able to control the infection (Figure 4A; red dashed lines), and the total population size decreases dramatically to 50% of the carrying capacity (i.e., maximal population size), confirming the results from the probabilistic model. However, after centuries of infection, the fraction of recovered individuals increases, and with it the total population size, indicating a recovery of the population. This observation is consistent in all ten simulations we performed for iNKRs (Figures 4A,C).

To study how these individuals evolve to control an infection with a virus having an MHC-like molecule, we analyze the average specificity of the NKRs over time. We determine how many MHC molecules in the population can be recognized by each receptor, and normalize it by the number of total MHC molecules in the population (Figure 4E; red line). We observe that the specificity increases after the emergence of the decoy viruses. At the end of the simulations, each iNKR recognize <20% of the MHC molecules in the population, indicating that evolution selects for specific iNKRs.

We perform the same simulations and analysis for populations having only aNKRs. Compared to populations having iNKRs, the initial spread of the virus is somewhat impaired (Figure 4B; red solid lines). Already at the beginning of the infection, some individuals are able to control the virus (Figure 4B; red dashed lines). The population size decreases to 60% of the carrying capacity, and is therefore fitter than in those simulations considering only iNKRs (Figures 4A–C). Thus, aNKRs provide a better initial protection than iNKRs. Accordingly, the number of recovered individuals and thereby the total population increases rapidly, reflecting their fast recovery against viruses evolving decoy proteins.

The higher protection of aNKRs compared to iNKRs can be explained by the initial specificity. Because we initialize all populations with intermediate specificity, individuals carrying only aNKR are initially better protected (Figure 2). Nevertheless, aNKRs also evolve to be more specific (Figure 4E; black lines). At the end of the simulation, aNKRs recognize on average approximately 35% of all MHC molecules, and hence decoys in the population. Taken together, our agent-based model reveals that iNKRs need to be more specific than aNKRs to protect during an infection with a CMV-like virus, confirming the results from our probabilistic model.

Note that we do not explore all possible loci number in the agent-based model. To save computational time, we test the evolution of the specificity given a fixed loci number of NKRs. Populations carrying 10 NKR loci correspond to 20 NKRs in the probabilistic model, where the protection is maximal at very high specificity values for iNKR, and intermediate values for aNKR. These values correspond indeed to the specificity values that the populations evolve in our simulations. We carried out additional simulations for 5 and 15 NKR loci, the results of which confirmed the predictions of the mathematical model (results not shown).

### Populations having only aNKRs evolve a larger NKR polymorphism than populations with only iNKRs

Our probabilistic model predicts that the protection by iNKRs and aNKRs increases with the number of receptors per individual (Figure 2), because a large receptor number increases the chance of a host carrying very specific NKRs to have licensed receptors. This observation suggests that heterozygous hosts should have an advantage over homozygotes. We therefore hypothesized that heterozygous advantage must be selecting novel NKRs in our agent-based model, driving polymorphism of NKRs in the population.

To measure the polymorphism at population level, we use the Simpson’s reciprocal index (SRI, see Materials and Methods). The SRI is a diversity measure that is equal to the total number of NKRs if they are equally distributed in the population, whereas the SRI is lower than that in a population where some alleles dominate (34).

The initial polymorphism of aNKRs (i.e., SRI = 10) increases over time (Figure 4G; black line), reflecting that a high number of aNKRs provides indeed an advantage. But surprisingly, this is not the case for iNKRs, where the diversity decreases to SRI = 7. Because the agent-based model considers a limited number of MHC molecules in the population, the specificity that the iNKRs evolve in the simulations is lower than that observed in the analytical model (i.e., *p_i_*_,simulations_ ≈ 0.18 compared to *p_i_*_,analytical_ = 0.10) (see Figure 2). With this specificity value that is slightly lower than expected, all haplotypes tend to cover the entire (finite) MHC space, making it possible to have at least one licensed receptor. As a result, these populations can be well protected with a lower number of receptors. Therefore, there is little heterozygous advantage in populations having only iNKRs, resulting in a low level of polymorphism. Thus, the agent-based model finds a different solution for an optimal protection: it evolves contracted haplotypes (i.e., composed only of seven receptors) with slightly less specific iNKRs than expected.

### Protection depends on the number of MHC loci

To confirm our results concerning the dependency on MHC loci number, we also perform simulations with individuals having two MHC loci. An increasing number of MHC loci has a large effect on the protection provided by aNKRs. Although these populations are initialized with intermediate specific NKRs, the initial protection is lower than in the population carrying only one MHC locus (Figure 4D). For better protection, a higher specificity is required, and the selection for more specific aNKRs is stronger in these simulations (Figure 4F). As a result of the higher specificity, a larger number of receptors per haplotype are necessary to become licensed and to recognize the foreign decoy molecules. Therefore, the advantage of heterozygotes over homozygotes is larger in these populations, resulting in a higher degree of polymorphism (Figure 4H).

The protection and evolution of iNKRs is less sensitive to the number of MHC loci per individual. Like in the simulations considering one MHC locus, we observe a recovery of the population as more specific receptors are evolving (Figures 4D,F). Because the total number of MHC alleles is larger in populations having two MHC loci, more iNKRs per haplotype are necessary to have at least one licensed receptor. Hence, the total SRI score is higher in these simulations, than in the case of single MHC locus (Figures 4G,H; red line).

### Basin of attraction: Mixed haplotypes containing a majority of aNKRs

Finally, we performed simulations of populations having both iNKRs and aNKRs, in which we allow for the evolution of the specificity and also the receptor type. The initial specificity values for both receptor types was intermediate (i.e., *p* ≈ 0.4) and we initialized the genotypes with a random number of activating and inhibitory receptors.

**Figure 5:**
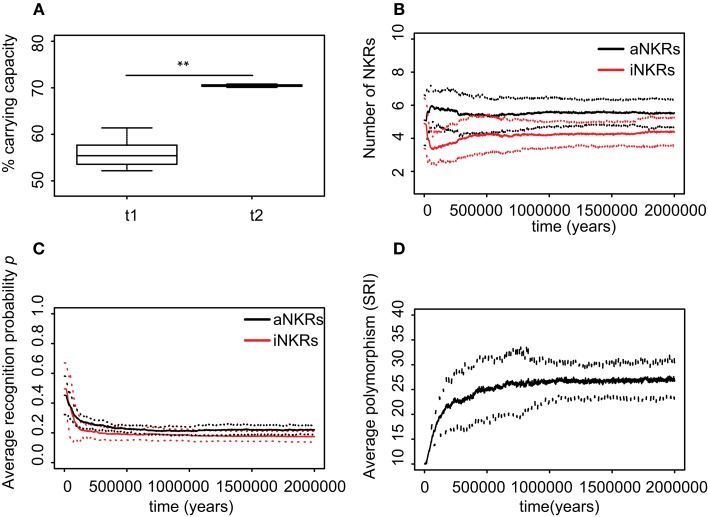
**Mixed haplotypes offer the highest protection**. A host population having iNKRs and aNKRs was inoculated with a wild-type virus after a period of *t* = 5000 years; we allowed for the evolution of decoy viruses 10,000 years after the initial epidemic (i.e., *t*_1_). **(A)** The population size during the initial spread of decoy viruses (*t*_1_) is lower than that at the end of the simulations (i.e., *t*_2_ = 3 million years), indicating that over time, the population recovers from the viral infection. **(B)** The initial haplotype is composed of five iNKRs and five aNKRs. The number of aNKRs and iNKRs per haplotype varies over time, resulting in a selection for haplotypes with a larger activating potential. **(C)** The probability of NKRs recognizing any random MHC molecule in the population, decreases over time, indicating that more specific receptors are being selected for. **(D)** The degree of NKR polymorphism (expressed as the SRI score) increases in time, as a result of the heterozygote advantage due to the evolved higher specificity. Averages are taken out of ten different simulations. In **(A)**, the boxes represent the interquartile range, and the thick horizontal lines the median (**represent *p* values <0.005, and were calculated using the Mann–Whitney *U* test). In **(B–D)**, the solid lines represent the average out of ten simulations, and the dashed lines are the standard deviation.

After the appearance of decoy viruses, the populations suffered similar effects to those having only iNKRs and aNKRs. The population size decreases dramatically at first, and with time it recovers. The final population size is higher than in the simulations considering only one type of receptor, approaching 70% of the carrying capacity (Figure 5A) because mixed haplotypes protect better than only one type of receptors. At the end of the simulations, we observe more aNKRs than iNKRs per haplotype (Figure 5B), i.e., the final haplotypes are composed on average of 6 aNKRs and 4 iNKRs. In agreement with the predictions of the analytical model, both receptor types evolve high specificity (i.e., *p* ≤ 0.35), and a high polymorphism (Figures 5C,D). Summarizing, the agent-based model confirms the prediction of the probabilistic model.

## Conclusion and Discussion

Our mathematical model predicts the optimal protection level provided by inhibiting and activating NKRs against viruses expressing MHC-like molecules. Haplotypes composed only of iNKRs detect the viral infection within a small range, requiring high specificity and large haplotype size. In contrast, the maximal protection is reached for intermediate specificity values and at a smaller haplotype size in individuals having only aNKRs. Mixed haplotypes, i.e., haplotypes carrying both iNKRs and aNKRs offer the highest protection.

All these results are dependent on the number of MHC loci per individual. With increasing MHC loci, aNKRs lose their ability to become licensed and thus provide little or no protection. In contrast, haplotypes composed only of iNKR have a higher chance of having licensed receptors when the number of MHC loci is increased. In this case, the protection level is maximal already with a contracted NKR haplotype. Thus, there seem to be several combinations of MHC–NKR genotypes that provide maximal protection. A high protection is reached with a simple MHC complex and a high number of NKR genes. With increasing complexity of the MHC, a contracted NK complex is sufficient to render protection. These last results are particularly interesting, as they provide a possible explanation of the differences in KIR and MHC gene content across primate species (35), and the expansion of new KIR lineages corresponding to the contraction of the MHC gene complex, thus illustrating the co-evolution of MHC class I and KIRs (36).

The model described here is inspired by viruses evolving decoys. However, its main outcome, namely the requirement for specificity, might be more general than the defense against such decoy viruses. Studies have shown that viral infections can change the repertoire of peptides presented by MHC class I molecules (37), and that these different peptides affect the NKR–MHC interactions, perturbing the binding of iNKRs and leading to NK cell activation (38). In such cases, specific recognition of the changes in peptide repertoire by NK cells seems advantageous for the host. Also, the specificity ranges obtained in our model for mixed haplotypes (Figure 3E) are similar to those observed in reality, with iNKRs having a specificity of 0.2. This corresponds to the four mutually exclusive epitopes that have been detected so far for inhibitory KIRs in humans: HLA-A11, -Bw4, -C1, and -C2.

The exact role of aNKRs remains intriguing. Since only a few aNKRs tend to recognize MHC class I molecules (39), we speculate that aNKRs could specifically recognize new ligands expressed upon viral infection (e.g., decoy molecules or stress ligands). Our model predicts that to face the challenge of not recognizing self but specifically recognize foreign antigens, aNKRs do not need to be so specific. Indeed, the haplotype providing the highest protection is a combined haplotype composed of more aNKRs than iNKRs, which disagrees with the most primate KIR haplotypes (36). Most primate NKRs are inhibitory, and activating receptors have been linked to selection pressure induced by reproduction (36). Our model predicts that aNKRs should evolve to an intermediate specificity upon CMV-like infections. However, not many activating ligands have been identified yet, and it remains puzzling what other roles aNKRs might play.

The expansion of NKR superfamilies, presumably in order to gain resistance against pathogens, illustrates the high evolutionary complexity of NKRs. We aimed to fully understand the effects of a single possible driving force of this evolutionary process, namely that of a viral encoded MHC-like molecule. Therefore, we focused on modeling only the evolution of NKRs in a simple model, which requires simplifying assumptions. For instance, we fixed MHC polymorphism despite the evidence of the co-evolution between MHC class I and KIRs (35, 36). Given their different evolutionary timescales, i.e., that MHC molecules are older than both Ly49 and KIRs, we chose to model the expansion and contraction of NKR systems within an already existing MHC diversity. Additionally, we assumed that decoy viruses down-regulate the expression of all MHC molecules in the host. Even though we do not expect selective MHC down-regulation to affect the evolution of aNKRs (since activating receptors cannot detect missing-self), the evolution of iNKRs might be affected because more licensed iNKRs will be necessary to recognize a virus that down-regulates only one of the host’s MHC-I molecules. Note that if the licensed repertoire of iNKRs is larger, these receptors should be even more specific to avoid being “fooled” by the decoy molecule. The exact effect of selective MHC down-regulation on the specificity of iNKRs is an open interesting question, which we are currently working on.

Other simplifying assumptions were also necessary, such as considering a global NKR repertoire and ignoring the synergy between NKRs or the direct interaction between immune cells. Additionally, we ignored mutational operators that conserve similarity between pre- and post-mutation receptors (e.g., point mutations), as we only model mutations that significantly change receptor functionality. Including point mutations, did not affect the results qualitatively (results not shown), however a longer evolutionary time was necessary to approach the same solution of specificity. Overall, since our main results are of a qualitative nature, it seems unlikely that relaxing any of these assumptions would affect our main results. Note also that our agent-based model is inspired on humans and KIRs, with the advantage of having realistic parameters for processes like birth and death. However, the model can be generalized to other species, and qualitatively it represents a model of the evolution of the expansion of the NKR complex.

All our analytical results were consistent with the agent-based model and our analysis allowed us to quantify the protection against an infection for both receptor types. It confirmed our previous results that iNKRs should become specific enough (28). Our new approach has shed light into the possible contribution that each receptor type confers upon infection, and allowed us to conclude that mixed haplotypes render the best protection.

## Materials and Methods

### Agent-based model

The agent-based model consists of two types of actors (hosts and viruses) and three events (birth, death, and infection). This model is virtually identical to the one published in Ref. (28). Briefly, we screen all hosts in a random order during each time step of 1 week, and confront them to one of the randomly chosen events. Hosts age over time and at the end of each time step, their age, infection state, and type of infection is updated. This cycle is repeated for two million years to simulate long term evolution. All model parameters are given in Table 1.

We model simplified diploid individuals, carrying gene complexes for NKR and MHC class I. For simplicity, we consider 15 MHC alleles per locus, resembling the most common HLA alleles in the European population (40). NKRs and their ligands are modeled with randomly generated bit strings as a simplified representation of amino acids (41). If the longest adjacent complementary match between two strings exceeds a threshold *L*, we allow for the receptor to interact with its ligand. Thus, the threshold *L* determines the specificity of each receptor: a receptor with a small *L* value will be very degenerate and the probability of a random NKR to recognize a random MHC molecule will be *p* ≈ 1. In contrast, a receptor with a large *L* value will be specific, and accordingly, the probability of this receptor binding any MHC molecule in the population will be *p* ∼ 0 [for a detailed description, see Ref. (28)].

### Receptor types

In the present model, we allow for the evolution of aNKRs. When a novel NKR is generated, a random *L* value between 1 and 16 is assigned to it, and its type (i.e., whether it is activating or inhibitory) is also randomly chosen. Thus, each receptor has its particular specificity and functionality. Receptors with *L* values larger than 13 will usually not recognize any MHC molecules in the population, and are typically not functional. Genes encoding such non-functional NKRs are considered to be pseudogenes. Haplotypes containing pseudogenes are effectively shorter than haplotypes composed of fully functional NKRs. Thus, we can model the contraction and expansion of the NKR gene complex.

### Viral infections

In our simulations, we consider one wild-type virus and several “decoy viruses,” i.e., viruses expressing MHC decoys. Each virus comes with a viral load, which is implemented as an increase of the host’s death rate, *VL_i_* depending on the infection state *i* (see Table 1), and a probability of clearing the infection *p_wt_* and *p_dec_*_,_*_s_* for the wild-type and the decoy viruses, respectively. A decoy virus down-regulates the expression of all MHC molecules in that host, and encodes one MHC-like molecule. The evolution of decoy molecules is modeled by allowing the virus to adopt a randomly selected MHC molecule from its host with a rate μ*_v_*. The virus keeps this decoy for the rest of the simulation. Because we fix the MHC polymorphism to 15 alleles per locus, the maximal number of decoy proteins that can evolve in the population is 15 for the simulations considering 1 MHC locus, and 30 for those considering two MHC loci.

We consider different levels of protection against a decoy virus, depending on the success of the virus to escape the NK cell response, *s*. If at least one of the licensed iNKR binds to the decoy molecule, there will be an inhibitory signal, the host will not be able to detect “missing-self,” and the decoy virus will be successful. Similarly, if none of the licensed aNKRs recognizes the decoy molecule, the decoy virus will evade the NK cell response. Thus, none of the iNKRs or at least one aNKRs should bind the decoy molecule to render protection (Table 2). We model the immune escape by setting the probability of clearing the infection to zero, letting the host become chronically infected. In the case that a decoy is not successful, the host will be able to detect “missing-self.” Since this virus is nevertheless able to evade the response from T cells (due to the MHC down-regulation), the probability of clearing the infection is lower than that of the wild-type virus (*p_wt_* = 0.85). The resulting probability of clearing the infection is described by:

(5) $$p_{dec,s}=\left\{\begin{array}{cc} 0,\; & \mathrm{if}\;s=0\;\left(\mathrm{successful}\;\mathrm{decoy}\right)\\ 0.5,\; & \mathrm{if}\;s=1\;\left(\mathrm{unsuccessful}\;\mathrm{decoy}\right).\\ \; \end{array}\right.$$

The rest of the parameters defining the infection dynamics and immune escape of the decoy viruses (i.e., time of infection, immunity time, and transmission probabilities) were set like in Ref. (28) and are described in Table 1.

### Mutation

During each birth event, NKRs undergo mutation with a probability, μ. To decrease computation time, we model mutation by randomly creating a new receptor with its particular specificity and signaling type. We do not consider other mutational operators, e.g., point mutations, recombination, deletion, or duplication.

We first perform simulations where only the specificity can evolve (i.e., a random value *L* is assigned to each new receptor), while the receptor type remains fixed. Hereby, we are able to compare what the basin of attraction for the specificity will be, if a population has only aNKRs or only iNKRs. We also simulate populations with mixed haplotypes, by allowing the receptor type to mutate.

### NK cell education

During the birth event, an NK cell education process takes place. Like in our probabilistic model explained above, iNKRs which recognize *at least one* of the MHC molecules within one individual, and aNKRs that fail to recognize *all* of the MHC molecules within the host, are set to be licensed. In our model, only the licensed repertoire of NKRs will participate in an NK cell response (Figure 1).

### Model initialization

The model is initialized with a host population of 4500 hosts, with random ages between 1 and 70 years corresponding to a uniform age distribution. After approximately 10 host generations, this age distribution corresponds to more modern age distributions with the majority of individuals having an age between 15 and 60.

At the start of every simulation, a gene pool for MHC alleles is generated, the size of which depends on the number of MHC loci per individual. It consists of 15 alleles in simulations considering one MHC locus per individual, and of 30 alleles in those simulations considering two MHC loci per individual. To create the initial genome of each individual, MHC genes were randomly drawn from the pool, while ten NKRs with intermediate specificity (2 ≤ *L* ≤ 4, i.e., *p* ≈ 0.4) were generated. Thus, the initial haplotypes did not contain any pseudogenes. In the simulations considering mixed NKR haplotypes, the initial genes can be both activating and inhibitory. The type of each receptor was randomly chosen as explained above, resulting in approximately 50% of the receptors being activating. All individuals were initialized with the same NKR haplotype, but with different MHC genes.

### Genetic diversity

The Simpson’s Index is a measurement of diversity that can be interpreted as the probability that two randomly chosen receptors from two random hosts in the population are identical (34). The reciprocal of the Simpson’s Index defines a “weighted” diversity. The SRI was calculated as follows: $\text{SRI}=\frac{1}{\sum_{i=1}^{N}\;f_{i}^{2}},$ where *f_i_* is the frequency of the receptor *i* over all NKRs in the population, and *N* is the total number of unique NKRs.

### Implementation

The model was implemented in the C++ programing language. We considered populations with haplotypes composed of only aNKRs, only iNKRs, or both. In every scenario, we compared the effects of one or two MHC loci per individual. For each of these settings, we performed ten simulations for 2 million years. The code is available upon request.

## Conflict of Interest Statement

The authors declare that the research was conducted in the absence of any commercial or financial relationships that could be construed as a potential conflict of interest.

## Appendix

### Global repertoire of licensed NKRs

Instead of considering individual NK cells expressing a tuned set of NKRs, we model a global repertoire of “licensed” NKRs in each host. Hereby, we assume that the expression of at least one “licensed” receptor is sufficient to educate NK cells, and that those functional NK cells can become activated upon viral infection. This assumption needs further explanation.

If an aNKR recognizes some self-molecule (or self-MHC), all developing NK cells expressing that receptor should additionally express at least one iNKR recognizing self to prevent self-reactivity. As a consequence, all NK cells expressing that aNKR should never become activated, even if a virus encodes a ligand which engages that aNKR. Therefore, we call such an aNKR “unlicensed”: it will never contribute to detect a viral infection. In contrast, if an aNKR that does not recognize any self-molecule, all developing NK cells expressing that receptor will not be influenced by it, and these cells will express other iNKRs and aNKRs that balance their self-reactivity. NK cells expressing that aNKR will become activated when a virus expressing its ligand engages that aNKR. Therefore we call such an aNKR “licensed.” Any NK cell expressing this aNKR should breach its activation threshold, expand, and protect, when the ligand is present.

Now consider an iNKR that recognizes some self-MHC. All developing NK cells expressing that receptor will be tuned to balance its self-reactivity. As a consequence, all NK cells expressing that iNKR should become activated when a virus down-regulates this particular MHC. Therefore, we call such an iNKR “licensed”: it detects MHC down-regulation. However, for an iNKR that does not recognize any self-MHC, the developing NK cells expressing that receptor will not be influenced by that iNKR. Consequently, these cells will express other iNKRs and other aNKRs in order to balance their self-reactivity. Therefore, NK cells expressing that iNKR will not become activated by this iNKR when a virus down-regulates self-MHC. Therefore, we call such an iNKR “unlicensed”: it would never contribute to the detection of virus infected cells.

What happens with the NK cells expressing such an unlicensed iNKR if it happens to recognize something else on virus infected cells, e.g., a decoy? Then the iNKR should deliver an extra inhibitory signal to those cells. Consequently, these NK cells cannot expand and protect, even if their other iNKR detect MHC down-regulation, or if their other aNKR detect a new ligand. Hence, such an unlicensed iNKR will not protect, and can be considered to be non-functional at the repertoire level.
