# Corrigendum: Quantifying the Protection of Activating and Inhibiting NK Cell Receptors during Infection with a CMV-Like Virus

**DOI:** 10.3389/fimmu.2014.00663

**Published:** 2015-01-05

**Authors:** Paola Carrillo-Bustamante, Can Keşmir, Rob J. De Boer

**Affiliations:** ^1^Theoretical Biology and Bioinformatics, Department of Biology, Utrecht University, Utrecht, Netherlands

**Keywords:** agent-based modeling, NK cell receptors, evolution, CMV infection, models, theoretical

We found a minor implementation error in the NK cell education process of our agent-based model. Fortunately, this hardly affected our main results and conclusions. The main difference lies in the polymorphism of iNKRs, as our new results show that it increases substantially over time similar to that of aNKRs. For reasons of accuracy and reproducibility, we here provide the corrected Figures and paragraphs (underlined).

## Populations Having Only aNKRs Evolve a Larger NKR Polymorphism than Populations with Only iNKRs

Our probabilistic model predicts that the protection by iNKRs and aNKRs increases with the number of receptors per individual (**Figure 2**), because a large receptor number increases the chance of a host carrying very specific NKRs to have licensed receptors. This observation suggests that heterozygous hosts should have an advantage over homozygotes. We therefore hypothesized that heterozygous advantage must be selecting novel NKRs in our agent-based model, driving polymorphism of NKRs in the population.

To measure the polymorphism at population level, we use the Simpson’s reciprocal index (SRI, see Material and Methods). The SRI is a diversity measure that is equal to the total number of NKRs if they are equally distributed in the population, whereas the SRI is lower than that in a population where some alleles dominate (34).

The initial polymorphism of aNKRs (i.e., SRI = 10) increases over time (Figure 4G black line), reflecting that a high number of aNKRs provides indeed an advantage. Similarly, the SRI score of iNKRs increases over time. Because each evolved iNKRs recognizes on average one MHC molecule in the population, there is selection for haplotypes that do not overlap in the MHC molecules they recognize. Thus, the heterozygote advantage is large in these populations, driving the diversity of iNKRs.

**Figure 4:**
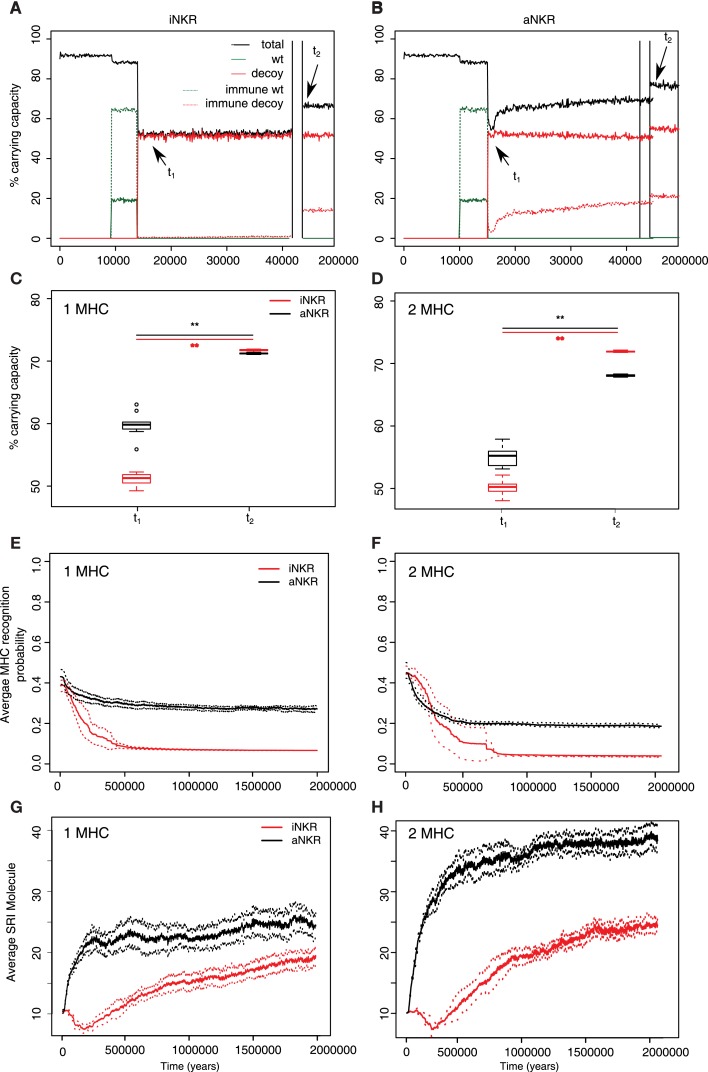
**Agent-based model confirms probabilistic model**. **(A)** A host population having only iNKRs was inoculated with a wild type virus (green lines) after a period of *t* = 5000 years (green solid lines show the chronically infected individuals, and the dashed lines the immune individuals). 10,000 years after the initial epidemic (i.e., *t*_1_), we allowed for the evolution of decoy viruses (red solid lines show the chronically infected individuals, and the dashed red lines the immune individuals). During the wild type infection, most individuals recover (dashed green line). In contrast, almost none of the individuals are initially capable of clearing a CMV-like infection (red dashed line), resulting in a large decrease of the total population size (black line). **(B)** A host population having only aNKRs is initially better protected against decoy viruses, resulting in a higher fraction of the population clearing the infection, and a lower decrease of the total population size. **(A,B)** show single representative simulations. **(C)** The average population size during the initial spread of decoy viruses (*t*_1_) is lower than that at the end of the simulations (i.e., *t*_2_ = 3 million years), indicating that over time, the populations learn to cope with the viral infection. Individuals in simulations considering only aNKRs (black) are initially better protected than those in simulations considering only iNKRs (red). In these simulations, all hosts carry only one MHC locus. **(D)** The initial advantage that aNKRs have over iNKRs receptors decreases in simulations considering two MHC loci per individual. **(E)** The probability of iNKRs recognizing any random MHC molecule in the population decreases over time (red line), indicating that more specific receptors are being selected for. In contrast, aNKRs (black line) do not evolve such high degree of specificity. **(F)** aNKRs evolve to become more specific in simulations where individuals have two MHC loci. **(G)** The degree of NKR polymorphism (expressed as the SRI score) increases in time, as a result of the evolved higher specificity. **(H)** SRI score in simulations considering two MHC loci. In **(C,D)**, the boxes represent the interquartile range, and the thick horizontal lines the median out of ten simulations (**represent *p* values <0.005, and were calculated using the Mann–Whitney *U* test). In **(E–H)**, the solid lines represent the average out of ten simulations, and the dashed lines are the SD.

**Figure 5:**
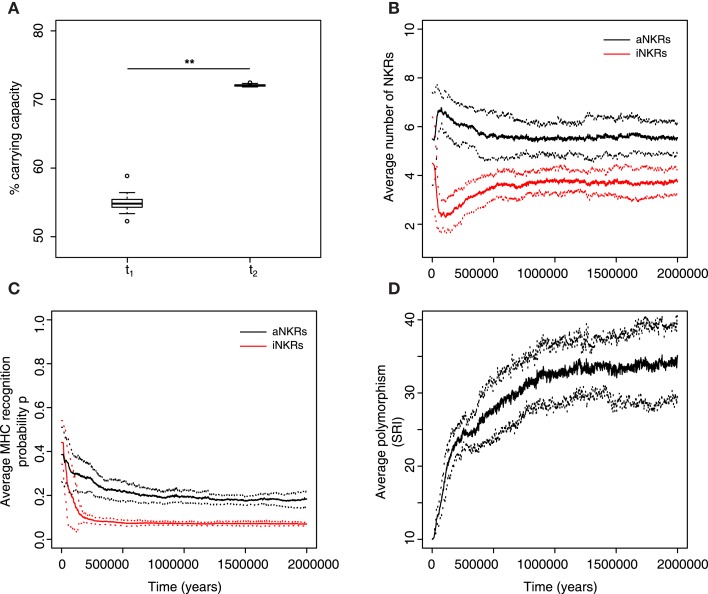
**Mixed haplotypes offer the highest protection**. A host population having iNKRs and aNKRs was inoculated with a wild type virus after a period of *t* = 5000 years; we allowed for the evolution of decoy viruses 10,000 years after the initial epidemic (i.e., *t*_1_). **(A)** The population size during the initial spread of decoy viruses (*t*_1_) is lower than that at the end of the simulations (i.e., *t*_1_ = 3 million years), indicating that over time, the population recovers from the viral infection. **(B)** The initial haplotype is composed of five iNKRs and five aNKRs. The number of aNKRs and iNKRs per haplotype varies over time, resulting in a selection for haplotypes with a larger activating potential. **(C)** The probability of NKRs recognizing any random MHC molecule in the population, decreases over time, indicating that more specific receptors are being selected for. **(D)** The degree of NKR polymorphism (expressed as the SRI score) increases in time, as a result of the heterozygote advantage due to the evolved higher specificity. Averages taken out of 10 different simulations. In **(A)**, the boxes represent the interquartile range, and the thick horizontal lines the median (**represent *p* values <0.005, and were calculated using the Mann–Whitney *U* test). In **(B–D)**, the solid lines represent the average out of ten simulations, and the dashed lines are the SD.

## Protection Depends on the Number of MHC Loci

To confirm our results concerning the dependency on MHC loci number, we also perform simulations with individuals having two MHC loci. An increasing number of MHC loci has a large effect on the protection provided by aNKRs. Although these populations are initialized with intermediate specific NKRs, the initial protection is lower than in the population carrying only one MHC locus (Figure 4D). For better protection, a higher specificity is required, and the selection for more specific aNKRs is stronger in these simulations (Figure 4F). As a result of the higher specificity, a larger number of receptors per haplotype is necessary to become licensed and to recognize the foreign decoy molecules. Therefore, the advantage of heterozygotes over homozygotes is larger in these populations, resulting in a higher degree of polymorphism (Figure 4H).

The protection and evolution of iNKRs is also sensitive to the number of MHC loci per individual. Like in the simulations considering one MHC locus, we observe a recovery of the population as more specific receptors are evolving (Figures 4D,F). Hereby, the specificity evolved to even higher values, as the evolved iNKRs recognize on average <5% of all the MHC alleles in the population. Because of this high specificity and the larger number of MHC alleles in populations having two MHC loci, more iNKRs per haplotype are necessary to have at least one licensed receptor. Hence, the total SRI score is higher in these simulations, than in the case of single MHC locus (Figures 4G,H red line).

## Conflict of Interest Statement

The authors declare that the research was conducted in the absence of any commercial or financial relationships that could be construed as a potential conflict of interest.

